# The reform patient information sheet sub study - an embedded trial evaluating the enhancement of patient information sheets to improve recruitment

**DOI:** 10.1186/1745-6215-16-S2-P87

**Published:** 2015-11-16

**Authors:** Sarah Cockayne, Joy Adamson, Peter Bower, Belen Corbacho, Caroline Fairhurst, Lisa Farndon, Kate Hicks, Anne-Maree Keenan, Peter Knapp, Sally Lamb, Lorraine Loughrey, Caroline McIntosh, Hylton Menz, Anthony Redmond, Jo Rick, Sara Rodgers, Wesley Vernon, Jude Watson, David Torgerson

**Affiliations:** 1 University of York, York, UK; 2 The University of Manchester, Manchester, UK; 3 NIHR Leeds Musculoskeletal Biomedical Research Unit, Leeds, UK; 4 Leeds Institute of Rheumatic and Musculoskeletal Medicine, Leeds, UK; 5 University of Oxford, Oxford, UK; 6 NUI Galway, Galway, Ireland; 7 La Trobe University, Bundoora, Australia; 8 Sheffield Teaching Hospitals, Sheffield, UK

## 

During recruitment, potential trial participants are usually given a written study patient information sheet (PIS). These are often long, complex and visually unappealing documents, which may have a negative impact on recruitment. Improving their readability by employing user testing, and their presentation by using graphic designers may improve patient understanding and aid recruitment.

We undertook an embedded randomised controlled trial within the NIHR-funded REFORM study, as part of the MRC START initiative, to evaluate whether enhancing PIS improves trial recruitment and retention. 6,900 patients due to be mailed a REFORM recruitment pack were randomised in a 1:1:1 ratio to receive one of three types of PIS: the original based on the NHS ethics template (n=2,298); an enhanced, user tested PIS (n=2,301); or a ‘template’ PIS which was developed using an enhanced PIS from another trial in a similar population (n=2,301).

193 participants (2.8%) were randomised to the trial: 62 (2.7%) in the control group; 63 (2.7%) in the user tested group; and 68 (3.0%) in the template group (OR: template vs control 1.10 (95% CI 0.77-1.56, p=60); user tested vs control 1.01 (95% CI 0.71-1.45, p=0.94); and user tested vs template 0.92 (95% CI 0.65-1.31, p=0.65)). Logistic regression analysis demonstrated that PIS allocation did not significantly predict recruitment (p= 0.33) or retention in the trial (p=0.83).

There was no evidence to suggest that enhanced PIS increased recruitment and retention rates to the REFORM trial. However, the number of patients randomised was low and we may be underpowered to detect a difference.

